# The impact of lifestyle restrictions on memory in older adults

**DOI:** 10.1371/journal.pone.0342458

**Published:** 2026-02-17

**Authors:** Jessica M.V. McMaster, Helena M. Gellersen, Saana M. Korkki, Jon S. Simons

**Affiliations:** 1 Department of Psychology, University of Cambridge, United Kingdom; 2 German Center for Neurodegenerative Diseases (DZNE), Magdeburg, Germany; 3 Aging Research Center, Karolinska Institute and Stockholm University, Solna, Sweden; Waseda University: Waseda Daigaku, JAPAN

## Abstract

Engagement in a variety of lifestyle activities, such as intellectual stimulation, social interaction, and physical exercise, is thought to be a key contributor to cognitive reserve, helping the brain compensate for age-related or pathological changes. An open question is whether restrictions on lifestyle activities, even if relatively brief, might have detrimental effects on cognition. The COVID-19 pandemic led to unprecedented restrictions on the kinds of lifestyle activities that have been shown to be protective against age-related cognitive decline. In the present study, we captured changes in lifestyle and memory of older adults across the pandemic. Long-term memory was assessed using a task which allows for the estimation of both retrieval success and memory precision, the latter being particularly sensitive to age-related changes. Memory was assessed before the pandemic in person, and during the pandemic using an online version of the task. Experiment 1 first verified that younger adults’ performance did not significantly differ between testing environments, validating pre- and post-pandemic comparison in older adults. Experiment 2 then demonstrated that while substantial declines in lifestyle engagement were observed during the pandemic in older adults, there was no significant correlation between these lifestyle changes and memory performance overall. However, when modelling retrieval success, lifestyle effects varied with dementia risk, consistent with cognitive reserve theory, as well as varying with depression. These findings highlight how different memory features are impacted by factors such as lifestyle, and support the proposal that heightened dementia risk may increase susceptibility to the impact of lifestyle changes.

## Introduction

There is a well-established link between lifestyle engagement (e.g., social and physical activity) and cognitive ability throughout the lifespan [e.g., 1], including evidence that active engagement in some lifestyle activities may confer resilience to the effects of aging on cognitive ability [2]. A relatively unexplored question is whether lifestyle restrictions might negatively impact cognitive performance in older adults.

Improvements in cognitive performance following positive lifestyle changes have previously been observed in intervention studies such as the Finnish Geriatric Intervention Study to Prevent Cognitive Impairment and Disability (FINGER; [3]), which involved older adults at increased risk of dementia. The intervention, which included dietary counselling, cognitive training, exercise training, and vascular risk management, had a positive effect across two years on cognitive performance. This included improvements in memory, but only when considering the more complex memory tasks which used longer test delays and tests of associative memory, highlighting the importance of using measures which are sensitive to age-related cognitive variability. Across intervention studies, including FINGER, MAPT [4] and PreDIVA [5], positive intervention effects were found particularly for individuals at high risk of dementia, which may suggest that these individuals are particularly susceptible to the effects of lifestyle changes. It remains an open question whether the positive effects of such interventions necessarily imply that reductions in lifestyle activities will have a measurable negative impact on cognition in older adults. A recent systematic review indicated that a substantially higher proportion of older adults with dementia experienced subjective worsening of cognitive impairment and mental health during the coronavirus (COVID-19) pandemic than older adults with healthy cognition [6]. Could the lifestyle restrictions experienced by many older adults, such as those implemented by governments around the world to reduce disease transmission during the pandemic, be a reason for this?

The association between dementia risk and the effects of varying lifestyle engagement is predicted by the notion of ‘cognitive reserve’, which is a resource thought to be developed through engagement in cognitively stimulating activities, such as those restricted during the pandemic [7]. It is theorised that cognitive reserve provides a buffer against the effects of brain changes due to both age and age-related diseases on cognitive performance, suggesting that reduction of cognitive reserve-related activities may have a particularly negative impact on older adults and those with underlying age-related neuropathology. The progressive relaxations of pandemic restrictions during 2020 and 2021 were often not applied to groups at increased risk of severe COVID-19 disease, including older adults (aged >70 years), which caused even greater periods of isolation than those experienced by other age groups. However, the duration of prior multidomain lifestyle interventions typically surpasses the duration of the pandemic-related restrictions, with the FINGER intervention assessed after two years [3] and other interventions far exceeding this duration [4,5]. Intervention studies have not yet determined how long interventions need to be to have a substantial positive (or, indeed, negative) effect on cognition [8] and the question is outstanding whether pandemic-related restrictions impacted cognition despite their relatively brief duration (lasting several months at a time in the UK) compared with multidomain lifestyle interventions (≥2 years).

In this study, we investigate the impact of pandemic-related lifestyle changes on memory, with a particular focus on older adults and the influence of dementia risk. Previous studies conducted to assess the impact of pandemic-related lifestyle changes on cognition have produced mixed findings. Evidence of cognitive decline has been found in some cases [9–12] but other studies have observed maintained or improved performance [13–16]. There have also been contrasting results within the same study when different cognitive measures are used [17–19]. These inconsistent findings may be due to the varying study designs used to address this research question.

The design of previous pandemic-focussed studies has, for example, varied in regard to whether they measure lifestyle changes explicitly or instead assume a universal impact of pandemic restrictions. A study involving the Lothian Birth Cohort 1936 [20] included a questionnaire to capture changes in aspects of lifestyle due to the restrictions, finding that memory changes during lockdown correlated with differences in wellbeing and social support. Vernuccio et al. (2022) observed a decline in cognitive, functional and neuropsychiatric symptoms after the COVID-19 pandemic, but did not capture individual-level effects of the pandemic, such as changes in lifestyle [12]. Therefore, it is not possible to distinguish changes due to pandemic-related restrictions and those due to time and increasing age. Perhaps somewhat unexpectedly, two studies of adults over the age of 50 years have found positive effects of the pandemic on engagement in protective lifestyle factors [21,22]. This emphasises the importance of including a lifestyle measure in such studies rather than assuming a universal decline in engagement. In the current study, participants completed the Lifetime of Experiences Questionnaire (LEQ; [23]) which involved reflection on both typical lifestyle activities as well as their lifestyle during the pandemic.

A consistent finding across pandemic-related studies is that cognitive change during this time relates not only to lifestyle but to other factors, including gender [18] and mental health [18,24–26]. Fiorenzato et al., (2021) reported that pandemic effects varied by gender, with females more likely to report worsening cognition and mental health [18]. In regard to mental health factors, a study of younger, middle-aged and older adults found that depression significantly impacted objective memory performance recorded across three timepoints in 2020, with the results indicating that the younger the participants, the more detrimental the effect of depressiveness was on memory change between time points [26]. In the current study, we capture gender as well as measures of depression and anxiety to further investigate the influence of these factors on memory change during the pandemic.

The choice of cognitive measure is a particularly impactful study design consideration. Studies lacking a pre-pandemic cognitive assessment often rely on retrospective and subjective questionnaires to capture changes in cognitive abilities [10]. However, these measures are limited by subjectivity and the accuracy of the responder’s memory for their past cognitive ability. Studies using both subjective and objective measures of cognition have found subjective cognitive decline related to the pandemic but a maintenance of objective cognitive ability [17,19]. Subjective memory decline could be due to a lack of variety in daily life experiences during the pandemic and lower frequency of memorable events compared with pre-pandemic times meaning memories of specific events during the pandemic are less available [27] or are remembered with less episodic detail [28]. However, the underlying memory processes may still be intact and, therefore, when directly tested using objective measures, performance may be maintained. Alternatively, the objective cognitive measures used previously may have not been sensitive enough to capture the cognitive changes reported through subjective assessments. In this study, we include both subjective and objective measures of cognition to gain further insight into how they may have been differently impacted by pandemic-related restrictions.

Prior studies have highlighted the importance of using sensitive cognitive measures when assessing the impact of lifestyle change (e.g., [3]). In the current study, we use a long-term memory (LTM) task which enables the estimation of both memory retrieval success and memory precision [29–31]. Binary retrieval success measures have been the focus of traditional memory tasks, however, memory precision is a continuous measure of the fidelity with which an event is remembered, and is more sensitive to age-related cognitive changes [32,33]. If pandemic-related lifestyle changes were detrimental to cognitive reserve, then older adults may demonstrate exacerbated effects of underlying age-related brain changes on memory precision which they were previously protected from. Furthermore, negative changes in retrieval success, which is less sensitive to the effects of age, may be particularly indicative of a substantial negative impact of pandemic-related lifestyle restrictions.

An online version of the LTM task was developed to assess memory during the pandemic when in-person testing was prohibited. The assumption that results obtained online are equivalent to those acquired in-person neglects potential variability in factors such as participant engagement, computer hardware, display properties and internet connection when testing online, which may impact data quality and validity [34]. To ensure that the LTM task test environment (in-person vs. online) did not confound the assessment of pandemic effects, an initial experiment compared the task performance of adults in-person and online.

The second experiment aimed to determine whether lifestyle changes during the pandemic had a measurable impact on older adults’ cognition, assessed using both objective and subjective memory measures. Participants from several in-person, pre-pandemic memory studies were invited to complete the online version of the task while pandemic-related restrictions were in effect. The in-person pre-pandemic studies had some variation in task format, but they all allowed for the estimation of both memory precision and memory retrieval success. We also assessed the influence of additional factors with evidenced links to cognitive performance, including dementia risk, gender, mental health, indicators of brain health, and age. Based on prior evidence, we predict that the impact of lifestyle change on cognition will be exacerbated in those with increased dementia risk, based on family history of dementia or lifestyle indicators (e.g., alcohol use, hypertension).

## General methods

### Participants

Both experiments were approved by the University of Cambridge Psychology Research Ethics Committee and all participants gave informed consent before participation. Participants in both experiments were fluent or native English speakers with normal or corrected-to-normal vision and were screened for neurological and psychiatric conditions.

### Memory task

In the encoding phase of each version of the memory task used in the present experiments, participants were shown a series of displays, containing objects overlaid on a background scene. After a delay, participants were shown a studied object and asked to recreate at least one of its features (e.g., location or colour) along a continuous response wheel [32,33,35,36]. See further details of the memory tasks used in each experiment below and in the S1 File.

### Data analysis

#### Non-parametric outcomes.

Response errors were calculated as the angular deviation between the target feature and the response (−180° to 180°).

#### Mixture modelling.

In the model fit to the data, participants’ responses are characterized by two sources of error. The first is the varying precision of successfully retrieved memories, represented in the model by a von Mises distribution centred on the target feature, with the width of the distribution corresponding to the participant’s memory precision, termed *SD*. This SD parameter was converted to *K* (*sd2k* function, https://www.paulbays.com/toolbox/) which describes precision as the concentration of the von Mises distribution to ease interpretation, as higher values indicate higher memory precision [29]. The second source of error is due to unsuccessful memory retrieval in which participants made random guesses. This is represented in the model by a uniform distribution. Retrieval success is calculated as *pT = 1 – proportion of trials within the uniform distribution*. This model is well-established in characterising performance in the LTM version of the current task [30,32,33] and is the best fitting model to the current data (see S1 File).

The model was fit to subject-level data using the Bayesian approach within the MemToolbox ([37]; memtoolbox.org). However, in instances where the model failed to converge across multiple participants, an alternative semi-parametric approach was used for all participants using the BAYSLAB toolbox (https://www.paulbays.com/toolbox/) to generate less sensitive but more robust parameter estimates. This approach involved fitting the model to the group-level data and generating a model-derived guessing threshold (see S1 File; [32,35,38]). Using this threshold, subject-level estimates of retrieval success were calculated as the proportion of responses within the cut-off value and estimates of precision were based on the standard deviation of the error values for responses within the cut-off value (multiplied by minus one so that higher values indicate greater precision).

Subject-level cognitive outcomes were deemed outliers if they were more than three standard deviations from the group mean.

#### Statistical analysis.

Across both experiments, Bayes factors were applied to estimate strength of evidence for the null (*BF*_*01*_) or alternative (*BF*_*10*_) hypothesis using the default prior in JASP (r = 0.707), with a Bayes factor >3 considered moderate evidence [39].

## Experiment 1

The first experiment assessed whether younger adults’ LTM performance was impacted by test environment (in-person vs online). The results of this experiment have important implications for the interpretation of findings from different testing environments.

### Methods

#### Participants.

**In-person study.** 30 younger adults were recruited between 2018 and 2021 for a study which involved completing the LTM task in person, in a laboratory setting (reported in: [32]).

**Online study.** 39 different participants were recruited in 2021, either through the University of Cambridge recruitment site, who were paid for their time (15 participants), or from the staff of our collaborator company, Aviva, who were remunerated using their company volunteering time (24 participants). Six participants were excluded for reporting a developmental, neurological or current psychiatric disorder diagnosis. One participant was excluded for less than fluent English proficiency. One participant was excluded for previously completing the in-person LTM task. The final sample included 31 participants.

**Comparison.** The demographic information for participants tested in-person and online is presented in Table 1. The groups did not differ significantly in age, gender, or years of education.

**Table 1:** Experiment 1 participant demographic information by test environment.

	In-person (*n* = 30) *Mean (SD)*	Online (*n* = 31) *Mean (SD)*	Diff *p-value (BF*_*01*_)
**Age**	25.23 (5.02)	23.94 (2.95)	0.61^†^ (2.79)
**Gender**			
Female	14 (47%)	13 (42%)	0.85^††^(4.18)
Male	16 (53%)	18 (58%)	
**Education** (years)	17.13 (3.04)	16.65 (2.58)	0.82^†^(3.31)

Where Mann-Whitney test was used this approach was also used for the Bayesian t-test with 1000 samples. ^†^ Mann-Whitney test applied as data displayed non-normality and/or unequal variances; ^† †^ Multinomial test of equal proportions. BF_01_ provides the strength of evidence for the absence of a test environment effect.

#### Memory task.

The in-person task [32] was programmed using MATLAB [40], whereas the online version used PsychoPy ([41], *pavlovia.org*). Online participants could only take part on a laptop/PC to minimise hardware variability and began the task by matching an image to the size of a credit card, held up to the screen, to maintain consistent stimuli sizing across participants.

#### Procedure.

**Fig 1:**
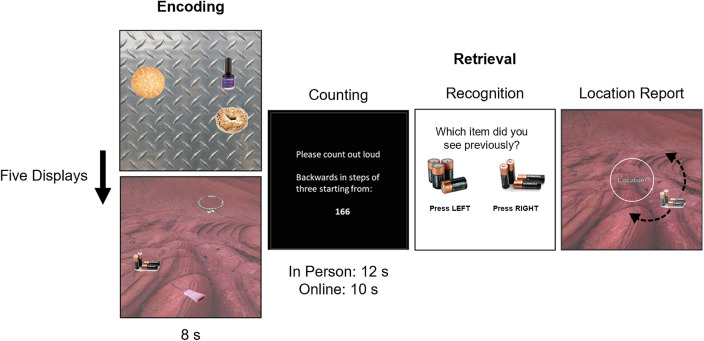
Task schematic used for Experiment 1 and online testing in Experiment 2. Includes stimulus images reproduced from the Konklab image repository (https://konklab.fas.harvard.edu) under a CC BY license and reproduced with permission from Konklab.

All participants first read instructions before completing a practice block of the task. Participants then began the full memory task where they viewed five displays for eight seconds each (Fig 1). The encoding phase was followed by a 12-second counting task to prevent rehearsal of memory-related information (10 seconds online to maintain engagement), followed by the retrieval phase which began with an object mnemonic discrimination question in which participants were instructed to distinguish between the previously presented target item and a simultaneously presented highly similar lure (174 objects in total; [42,43]). The side of the screen on which the target was presented was randomised across participants.

After participants indicated which object they had observed in the encoding phase, the item was then presented in a random position around the circle and participants adjusted its position to match the item’s studied location as precisely as they could using the arrow keys, submitting their response with the space bar. The task was self-paced, but after 15 seconds the central “Location” text turned red to encourage participants to make their response and reduce variability in response times. All objects within each display were tested sequentially in a random order. Overall, the task consisted of one practice block with four displays and five full blocks each consisting of five displays.

Taken together, training and test stimuli included 29 uniform background scenes and 87 pairs of everyday objects (Konklab image repository at https://konklab.fas.harvard.edu and Google Image Search). Each background scene was randomly allocated three target objects to generate 29 trial displays. Each object’s location on the background scene was randomly generated from circular positions with a minimum separation of 62.04° to prevent overlap. Displays were generated once, and participants viewed the same displays in a randomised order.

### Analysis

#### Group-level permutation analysis.

For the location responses, models were fit to group-level data to improve the robustness of the outcomes. To assess differences in memory performance, group assignments were shuffled across 1,000 iterations, maintaining original group numbers. In each iteration, the model was fit across all trials for each of the two random groups. Finally, a *p*-value was calculated based on the proportion of iterations where group differences in model parameters were greater than true differences.

#### Subject-level comparisons.

Differences in subject-level outcomes were assessed in each analysis by two-tailed t-tests, with Mann-Whitney tests conducted when assumption checks indicated possible violations of normality and/or equality of variances.

### Results

#### Non-parametric subject-level outcomes.

Mann-Whitney tests indicated no significant group differences in the proportion of correct mnemonic discrimination responses (W = 473.00, *p* = .91, BF_01_ = 3.53) or mean absolute localisation error (W = 443.00, *p* = .76, BF_01_ = 3.57) with moderate evidence in favour of no difference between groups (Table 2). Response time analyses for the mnemonic discrimination and localisation responses are provided in the S1 File.

**Table 2:** Memory performance by test environment.

Outcome	In Person (*n* = 30) *Mean (SD)*	Online (*n* = 31) *Mean (SD)*	Diff *p-value (BF*_*01*_)
***Semi-parametric*** ***(subject-level)***			
Retrieval Success	0.82 (0.15)	0.79 (0.19)	0.76^†^ (3.53)
Precision ^Δ^	−18.61 (3.81)	−19.21 (5.53)	0.63 (3.47)
***Model parameters*** ***(group-level)***	*Estimate (95% CI)*		**Diff prob (permuted)**
Retrieval Success	0.73 (0.70-0.75)	0.67 (0.65-0.70)	0.40
Precision (*K*) ^Δ^	13.87 (12.75-15.15)	15.63 (14.01-17.56)	0.35
***Non-parametric*** ***(subject-level)***			
Mean localisation error (absolute)	31.45 (17.53)	34.22 (21.02)	0.76^†^ (3.57)
Mnemonic discrimination	0.89 (0.06)	0.85 (0.12)	0.91^†^ (3.53)

ΔMore positive values indicate higher precision; ^†^ Mann-Whitney test applied as data displayed non-normality and/or unequal variances. Subject-level precision is calculated using the semi-parametric approach whereas group-level precision is a parameter estimated by fitting the memory model to the group-level data.

#### Model-derived metrics.

**Fig 2:**
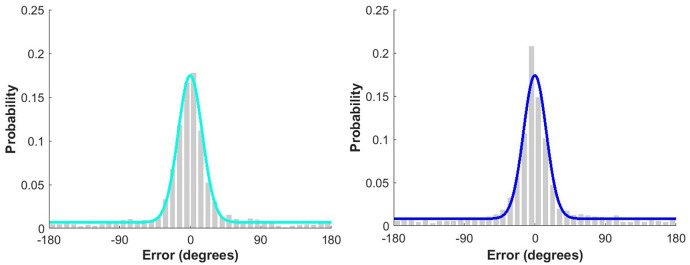
Model fit to response errors in the (left) in-person and (right) online studies.

**Fig 3:**
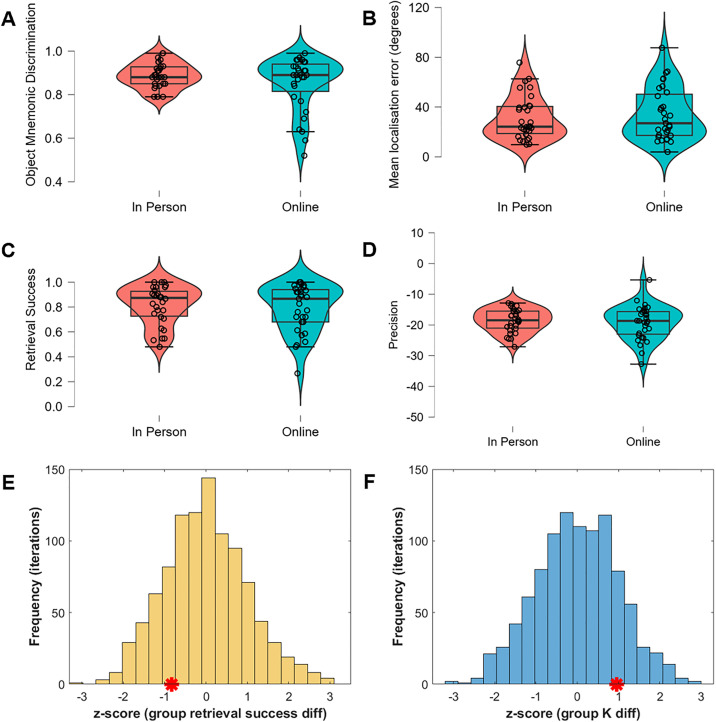
Memory performance by test environment. Plots (A-D) present the median as the central line, the first and third quartiles as the box edges, and the lines indicate the minimum and maximum values (within the range of the first and third quartiles multiplied by 1.5 times the interquartile range) for (A) mnemonic discrimination, (B) mean absolute localisation error, (C) semi-parametric retrieval success, and (D) semi-parametric precision. Plots (E-F) show the distribution of standardised group differences in (E) retrieval success and (F) precision (*K*) generated using a permutation procedure. The red star indicates the standardised actual difference in the model parameters (Online – In Person).

Models were fit to the in-person and online response error data to estimate memory precision and retrieval success (Fig 2) The results of the permutation analysis (Fig 3E-3F) indicated no significant differences between test environments in retrieval success (z-score: −0.83, p-value: 0.40) or memory precision (*K*, z-score: 0.95, p-value: 0.35).

Similarly, when estimating retrieval success and memory precision using the model-derived guessing threshold approach, there was moderate evidence for no group differences in the semi-parametric estimates of retrieval success (*W* = 486.50, *p* = .76, *BF*_*01*_ = 3.53) and precision (*t*(59) = 0.49, *p* = .63, *BF*_*01*_ = 3.47).

### Experiment 1 discussion

The memory task used here included an object mnemonic discrimination test, followed by a cued recall test assessing both retrieval success and memory precision. Across these memory outcomes, we found no significant performance differences between test environments, with moderate evidence (BF_01_ > 3) in favour of no differences. It appears, therefore, to be reasonable to compare online LTM performance with findings reported by our previous in-person studies that used similar tasks. A limitation of Experiment 1 is that it contained only younger adults, restricting the generalisability of these findings to older age groups. We focused on younger adults to minimise age-related cognitive variability, though online testing may pose distinct challenges for older adults. Even so, the current results offer preliminary evidence that the task performs comparably across test environments, supporting its use in Experiment 2, where online testing enabled the recruitment of participants who would otherwise be difficult to access during the COVID-19 pandemic.

## Experiment 2

Previous studies have established an association between engagement in certain activities (e.g., social, cognitive, physical) and resilience to age-related cognitive decline (e.g., [2]). These protective lifestyle activities were severely restricted during the COVID-19 pandemic. A question is whether such lifestyle restrictions, though relatively brief, had observable effects on cognition in older adults. Existing studies on the impact of lifestyle changes on cognition typically investigate the benefits of lifestyle interventions and suggest that individuals with dementia risk may in particular benefit from lifestyle changes [3–5]. There are also a number of life events such as transient or permanent injuries, loss of a partner or increased reliance on carers which could negatively impact a person’s lifestyle engagement. However, the possible detrimental effects of decreased lifestyle engagement are not often studied.

The objective of Experiment 2 is to determine the impact of pandemic-related lifestyle changes on memory, with a particular focus on older adults and the influence of dementia risk. This experiment involved older adults completing a LTM precision task, both in-person pre-pandemic (2016−20) and online during the pandemic (March 2021). The use of the online task to assess cognitive change is supported by the results of Experiment 1 which indicated no significant impact of test environment.

### Methods

Older adults from the Cambridge Memory Laboratory’s volunteer database (aged 60 + years), who had previously completed a version of the LTM precision task in person, were invited to participate in an online follow-up study.

#### Baseline studies (T1).

Older adults were recruited from the samples of three in-person studies which took place prior to the COVID-19 pandemic, between 2016 and early 2020. Full details regarding these studies, including their eligibility and exclusion criteria, are published elsewhere [32,33,36]. The three studies all involved versions of the LTM precision task, with minor differences in methods summarised in the S1 File. Across studies, participants scored in the healthy range when screened for cognitive impairment.

#### Follow-up study (T2).

The online follow-up study took place in March 2021, while severe pandemic-related lifestyle restrictions in England (where the majority of participants resided) were still ongoing. Of the 79 participants who completed the online memory task, ten participants were excluded due to unsuitable baseline data (e.g., precision scores not recorded). Several participants were excluded for conditions that were not reported at the time of their baseline study including uncorrected visual impairment (four participants), dyslexia (one participant) and certain health conditions (e.g., cancer, stroke, or traumatic brain injury) that were still impacting the participant (three participants). One participant was excluded for not completing multiple sections of the lifestyle questionnaire. Participants were excluded if they reported prior moderate-severe COVID-19 symptoms, resulting in one exclusion. Participants were not excluded for mild prior COVID-19 symptoms (three participants) but the effect of their inclusion was assessed in a sensitivity analysis (S1 File). Therefore, there were 59 datasets included in the full analysis (Demographic details in Table 3). One additional participant did not complete the occupation section of the questionnaire, so they are not included in analyses involving occupation scores. Further exclusion criteria for model-derived memory measures are outlined in the Analysis section below.

**Table 3:** Experiment 2 participant demographic information.

Variable	*(n = 59)* *Mean (SD)*
**Age (T2)**	72.98 (5.78)
**Gender**	
*Female*	39
*Male*	20
**Education (years)**	15.73 (3.75)
**Follow-up interval (years)**	2.76 (0.95)
**Depression (CESD-R10)**	7.14 (4.21)
**Anxiety (GAD-7)**	2.85 (2.96)
**LIBRA**	−0.16 (1.23)
**Family history of dementia**	
*Yes*	18
*No*	41
**Residential status**	
*Alone*	16
*With partner*	37
*With friend*	2
*With family*	3
*Other*	1

*Abbreviations*. LIBRA: Lifestyle for BRAin health [45].

As measures of depression and anxiety were included in the analysis, participants were not excluded for reporting these conditions, however, a sensitivity analysis was conducted with these participants excluded (S1 File).

#### Memory task.

Details of the in-person memory tasks can be found using the references included above, whereas the online version of the task is described in Experiment 1 (Fig 1). All of the tasks assessed LTM precision (*K*), testing memory for an object’s features. Here we focus on location memory where possible for consistency (otherwise memory for colour is used). Due to the similarity of tasks between studies, there may be practice effects, particularly for Gellersen et al., (2024;[32]) which was the closest study in time to the online follow-up and included the same memory displays as the online task. The impact of practice effects was explored by running a sensitivity analysis without the Gellersen et al., (2024;[32]) study and by including the time between sessions as a variable in the analysis.

#### Online questionnaire.

All participants completed an online questionnaire at T2 which was developed using Qualtrics (https://www.qualtrics.com) and captured a variety of health and lifestyle factors.

The questionnaire captured experience of COVID-19 symptoms as well as both physical health (e.g., Body Mass Index, family history of dementia, alcohol consumption) and mental health including scales for depression (CESD-R10; [44]) and anxiety (GAD-7; [45]). Information from the questionnaire was also used to assess brain health by calculating a Lifestyle for BRAin health (LIBRA; [46]) index for each participant, which is associated with dementia risk in middle-aged and older adults, and includes factors such as hypertension, hypercholesterolemia, diabetes, smoking, diet, and alcohol use (although not physical and cognitive activity in the current study due to overlap with the lifestyle questionnaire).

**Lifestyle change.** The Lifetime of Experiences Questionnaire (LEQ) was administered at T2 to measure lifestyle engagement and captures activities *specific* and *non-specific* to three phases of life: youth (13–30 years), mid-life (30–65 years) and late-life (65 years and older) [23]. Specific activities are those predominantly undertaken in one phase (e.g., education during youth, occupation during mid-life), whereas non-specific activities are common across all phases (e.g., physical activity). The scores from the participants’ most recent non-specific LEQ section were considered their “typical lifestyle” and these were compared to scores on the same questions when participants were asked to reflect on their lifestyle during the pandemic (scoring procedure outlined in the S1 File).

**Subjective memory.** The Prospective-Retrospective Memory Questionnaire (PRMQ, [47]) was used as a subjective measure of memory complaints. All participants completed this questionnaire at T2. The same questionnaire was applied in Gellersen et al., (2024;[32]), allowing us to measure change in subjective memory complaints in responders from that T1 study.

### Analysis

#### Memory task.

Memory outcomes were standardised across studies by calculating z-scores using the full sample of the original study (excluding four T1 participants and three T2 participants due to poor model fit). Retrieval success and precision scores (*K)*, based on fitting the memory model to subject-level data, > 3 SDs from the group mean were excluded based on pre-defined criteria, resulting in the exclusion of one participant from the retrieval success analysis and three participants from the analysis of precision (only two of these participants took part at T2). These exclusions resulted in 55 full datasets for the analysis of retrieval success and 54 full datasets for the precision analysis.

#### Statistical analysis.

***Did pandemic-related lifestyle changes influence memory performance?***

**Correlations.** We investigated the relationship between subject-level lifestyle change and memory change (T1-T2 difference scores) using Pearson’s correlation coefficients and Bayesian correlations (implemented in JASP, [48]). The Bayesian correlation analyses used a default stretched beta prior width of 1 which assumes that correlations in the range of −1–1 are equally likely.

***Is this relationship influenced by the risk of dementia?*** To investigate previous suggestions that dementia risk may be important in determining whether memory performance is influenced by lifestyle change, correlations were calculated separately for participants with and without a family history of dementia as well as for participants above and equal to/below the median LIBRA score. Bonferroni correction was applied for multiple comparisons, leading to α being set at 0.01. This analysis was not conducted for the subjective memory outcome as there were too few data points for meaningful conclusions to be made.

**Mixed-effects model.** To control for the effects of covariates and clustering of participants based on their baseline study, the relationship between lifestyle change and objective memory change was then assessed in a linear mixed-effects model using the *lme4* and *afex* packages in R [49–51]. This analysis again did not include subjective memory due to low data availability.

The model selection procedure involved the use of likelihood-ratio tests (LRTs, *p* < .05) and AIC values (Δ*AIC* > 2) to compare nested models. The time between sessions was included in all models due to its possible importance in controlling for practice effects and the main effects of baseline age and gender were assessed, and only retained in the model if they significantly improved model fit.

The effect of the baseline study was controlled for by including it as a random effect, although it was removed if it led to failed model convergence (see [52]) and the model was run as a linear regression using the *lm* function from the *stats* package [50].

For each final model, visual inspection was carried out to assess heteroscedasticity and the normality of residuals, and outlying data were assessed using Cook’s Distance [53]. Partial Cohen’s *f* squared values are reported for linear regressions [54], however there is not yet an agreed-upon approach to calculate standard effect sizes for the terms in mixed-effects models, so they are not included in this analysis.

***Is this relationship influenced by the risk of dementia?*** Family history of dementia and LIBRA score were individually introduced to the model both as a main effect as well as in a two-way interaction with lifestyle change to assess their contribution to model fit.

***Do other factors account for memory performance across time-points?***

Next, we conducted an exploratory analysis to investigate whether other factors, with evidenced links to memory, accounted for variance in memory performance both at each time point respectively as well as the change across time points. Once again, memory outcomes, including precision and retrieval success, were analysed using linear mixed-effects models. In addition to the variables already specified above, this analysis considered the effects of time point, occupation, education, anxiety score and depression score.

Once the maximal random-effects structure was identified, with the possible inclusion of baseline study and participant, the fixed effects were assessed by comparing a series of pre-specified, plausible alternative models. Time-point and the time between sessions were included within all of the models considered. When assessing the inclusion of additional variables, we included no more than two additional fixed effect terms and interaction terms did not involve more than three variables, to avoid overfitting and to ensure the interpretability of the results. Three-way interactions of continuous variables were assessed using the emtrends function from *emmeans* to compare differences across slopes [55]. All continuous variables were converted to z-scores to aid interpretation.

#### Sensitivity analyses.

Sensitivity analyses (detailed in the S1 File) were conducted to determine whether the results were robust to the exclusion of certain participant groups.

### Results

#### Responder vs non-responder analysis.

To investigate possible attrition effects, participants who completed the T2 study were compared with non-responders on demographic and cognitive factors using frequentist and Bayesian independent-sample t-tests. No significant differences were observed across factors between responders and non-responders (Table 4). The only comparison which reached trend-level significance (p = .08, uncorrected for multiple comparisons) was mean absolute error; however, the BF_01_ was close to 1, deeming the comparison inconclusive.

**Table 4:** Non-responder analysis.

Variable	Non-responder *(n = 92)*	Responders *(n = 69)*	p-value (BF_01_)
Age at baseline (T1)	71.13 (6.07)	69.91 (5.37)	0.19 (2.60)
Education (years)	16.92 (4.20)	16.52 (3.45)	0.95^†^ (5.19)
Female (%)	61.96	63.77	0.20^††^ (3.52)
Baseline performance (z-scores)			
MAE	−0.15 (1.01)	0.12 (0.98)	0.08^†^ (1.22)
Retrieval Success	***(n = 88)***	***(n = 68)***	0.23^†^ (3.80)
	−0.08 (0.99)	0.06 (1.04)	
Precision (*K)*	***(n = 87)***	***(n = 67)***	0.84^†^ (5.18)
	−0.01 (1.03)	−0.11 (0.86)	
MoCA*	***(n = 68)***	***(n = 42)***	0.68^†^ (4.77)
	28.18 (1.29)	28.26 (1.33)	

* Excludes Gellersen et al., (2024) study where the MoCA was not administered; ^†^ Mann-Whitney test applied as data displayed non-normality; ^† †^ Multinomial test of equal proportions. MAE: Mean Absolute Error, transformed so that higher values indicate better performance; Unless specified otherwise values indicate: Mean (SD); Where Mann-Whitney test was used this approach was also used for the Bayesian test with 1000 samples.

#### Lifestyle change.

To capture the effects of pandemic-related restrictions on lifestyle, item-level responses regarding typical lifestyle were compared with responses to the same items completed for lifestyle during the pandemic using frequentist and Bayesian paired-samples t-tests. Bonferroni correction was applied for multiple comparisons, leading to α being set at .003.

**Fig 4:**
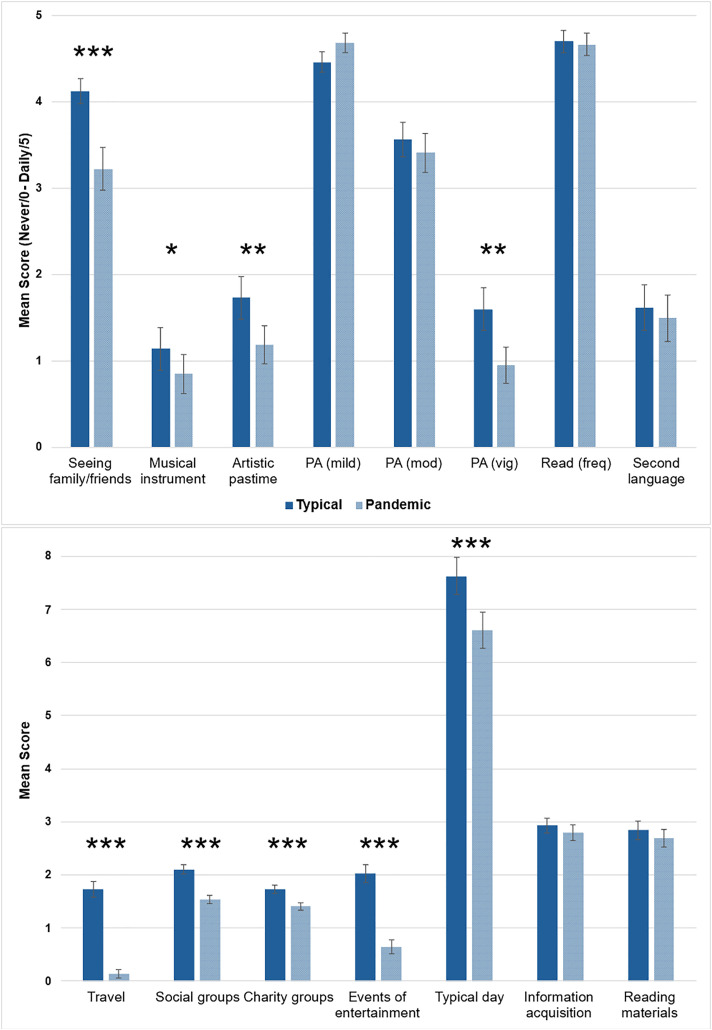
Lifestyle change. Top: Likert scale of 0-5 where 0 = never and 5 = daily activity. Bottom: Scoring procedure consistent with [23]. Higher scores indicate more engagement. After applying Bonferroni correction (α = 0.003), differences in playing a musical instrument and developing an artistic pastime were no longer significant. Abbreviations: PA, Physical Activity (mild, moderate, vigorous).

Overall, the pandemic-related lifestyle score (M = 28.90; SD = 7.53) was significantly lower than the score for participants’ “typical” lifestyle (M = 33.37; SD = 7.39; p < .0001, BF_10_ > 1000) indicating reduced engagement, although a strong positive correlation between the typical and pandemic-related lifestyle scores (*r*(57) = 0.86; p < .0001,BF_10_ > 1000) suggests that individuals with higher typical lifestyle engagement remained the more engaged during the pandemic. The item-level comparisons (Fig 4), using Wilcoxon signed-ranks tests, indicate that lifestyle change was widespread but not equivalent across activity types. In particular, social activities, vigorous physical activity, and activities that required leaving home (e.g., events of entertainment) were reduced, whereas some cognitive activities (e.g., reading) were maintained.

Certain activities, including travelling and involvement in social and charity groups, were substantially reduced; however, these responses were biased by the time frame being considered. For example, the number of places travelled to was naturally lower across the duration of the pandemic compared with their typical life phase which may have spanned up to 35 years. These items are removed from the analyses to minimise biases due to time frame differences.

#### Subjective memory failures (PRMQ).

In a subgroup of twenty-three participants who took part in the Gellersen et al., (2024;[32]) baseline study, the PRMQ was completed at both T1 and T2. Due to the sample size, multilevel modelling was not appropriate. Instead, a Wilcoxon signed-rank test found no significant difference in PRMQ score between T1 (M = 35.04, SD = 6.73) and T2 (M = 34.35, SD = 8.96; W = 160.00, *p* = .28, BF_01_ = 2.99). There remained no significant difference between time points when considering the prospective and retrospective subscores independently (p > .24, BF_01_ > 2.58).

#### Lifestyle and memory.

**Did pandemic-related lifestyle changes influence memory performance?**

**Correlations.** Across all participants, no significant associations were detected between subject-level lifestyle change and memory change, including memory precision (r(52) = 0.082; *p* = .56, BF_01_ = 4.98), retrieval success (r(53) = −0.005; *p* = .97, BF_01_ = 5.94) and subjective memory scores (r(21) = 0.132; *p* = .55, BF_01_ = 3.26). The same conclusions were reached when conducting correlations separately for participants with and without increased risk of dementia, based on family history of dementia or their LIBRA score (p > .14, BF_01_ > 1.23).

**Mixed-effects models.** When including change in memory precision as the outcome in a linear mixed-effects model, none of the considered variables met the criteria for inclusion. When change in retrieval success was the outcome in a linear regression, the only variable which significantly improved the model fit was the time between sessions, whereby a greater time between sessions was associated with a more negative change in retrieval success (b = −0.34, 95% CI [−0.55, −0.12], t(53) = 3.17, *p* = .003, partial f^2^ = .19).

**Do other factors account for memory performance across time-points?**

Following initial findings indicating no clear relationship between pandemic-related lifestyle change and objective memory change across participants, the contribution of other factors, to both memory performance at each time point and memory change (T1-T2), were assessed in an exploratory analysis.

**Fig 5:**
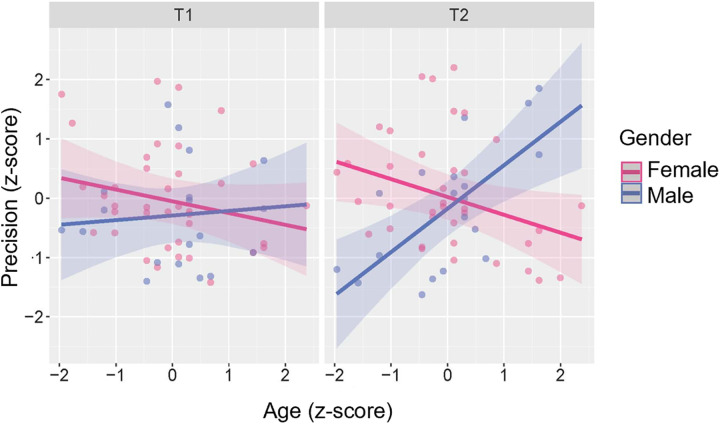
Marginal effects of precision model displaying three-way interaction effect. Age and precision values have been transformed to z-scores. Shading represents 95% confidence interval. A negative relationship between age and precision was observed for female participants at both time-points. In male participants, there was no marked age effect on precision at T1, however, at T2 there was a positive age effect on precision with older male participants performing better than younger male participants.

**Memory precision.** When including memory precision at each time-point as the outcome in a linear mixed-effects model with subject and baseline study as random effects, the best fitting model included age, gender, and time-point (model metrics supported the exclusion of the time between sessions). There was a significant three-way interaction between time-point, age, and gender (b = −0.19, 95% CI [−0.33,-0.05], t(102) = 2.64, p = .010) and visual inspection of the marginal effects (Fig 5) suggested that in female participants there was a negative relationship between (baseline) age and precision (Full model results are included in the S1 File).

The interaction between age, gender, and time point was explored further by modelling the memory precision of males and females separately. The best-fitting model for female participants included only age which had a significant negative effect on precision (b = −0.29, 95% CI [−0.53, −0.05], t(71) = 2.41, p = .019). In contrast, the best-fitting model for male participants included age and time-point with a significant interaction effect (b = 0.33, 95% CI [0.09,0.57], t(32) = 2.82, p = .008). The plotted marginal effects (Fig 5) suggest no substantial age effect in males at T1 on precision but a positive age effect on precision at T2 whereby older male participants performed better than younger male participants.

To determine whether the differing relationships between age and memory precision in males and females at T1 was unique to participants who returned at T2, the interaction effect between age and gender was also assessed in non-responders. A significant interaction effect was also observed in non-responders (b = −0.747, 95% CI [−1.18, −0.32], t(83) = 3.45, p < .001).

At T2, one possible driver of the positive age effect in males could be poor fit of the memory model in older males, however, visual inspection of the model convergence plots did not support this explanation. Alternatively, the age effect could be driven by attrition bias whereby older males who returned at T2 were particularly high-performing and resilient. This was investigated by running the responder versus non-responder analysis on males and females separately.

No significant differences were found in age, MoCA [56], years of education or baseline memory performance (retrieval success, precision) in males or females (*p* > .11, BF_01_ > 1.29). A trend-level significant difference was found for females in mean absolute localisation error (*p* = .06, BF_01_ = 1.56) with higher errors in non-responders although the comparison is considered inconclusive due to a BF_01_ close to 1.

**Change in memory precision.** We next focus on within-subject change in memory precision from T1 to T2, using a linear regression approach. Here, the best-fitting model which included occupation score and anxiety, was not robust to the exclusion of highly influential cases. In sensitivity analyses excluding participants who took part in the Gellersen et al., (2024;[32]) baseline study, the best-fitting model included age and gender, similar to the previous analysis of memory precision at each time point.

**Retrieval success**. When including retrieval success as the outcome in a linear mixed-effects model, the best fitting model included lifestyle scores (typical and pandemic-related) and family history of dementia as well as time-based effects (time-point and time between sessions). Removing influential data points meant some model terms were no longer significant. The following results exclude influential data points and are outlined in further detail in the S1 File.

**Fig 6:**
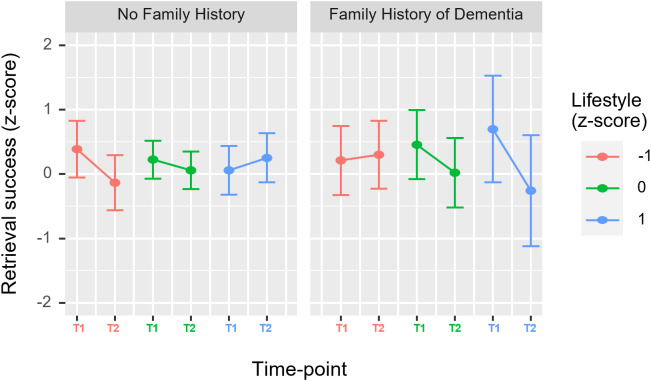
Marginal effects of retrieval success model displaying three-way interaction effect. All continuous values have been converted to z-scores. Bars represent 95% confidence interval. Results suggest that the lifestyle score associated with each time point (typical, pandemic) had a greater impact on memory performance at each time-point in participants with a family history of dementia. In participants with a family history of dementia, time-point had a more negative effect on retrieval success when participants had a higher lifestyle score, indicating higher lifestyle engagement.

This model produced a significant three-way interaction between time-point, lifestyle and family history of dementia (b = −0.22, 95% CI [−0.34, −0.10], t(82) = 3.61, *p* = .001). Visual inspection of the marginal effects (Fig 6) suggests that the retrieval success of participants with a family history of dementia, and therefore increased dementia risk, varied more substantially with differences in lifestyle and timepoint.

This interaction was explored further by modelling retrieval success separately for participants with a family history of dementia (N = 15) and without (N = 37). A significant interaction effect between lifestyle and time-point was present only in the model of people *with* a family history of dementia (b = −0.23, 95% CI [−0.37, −0.10], t(18) = 3.65, *p* = .002) but not in those without. Model metrics suggested that the three-way interaction (between lifestyle, time-point, and time between sessions) did not improve the fit of either model so was removed.

**Fig 7:**
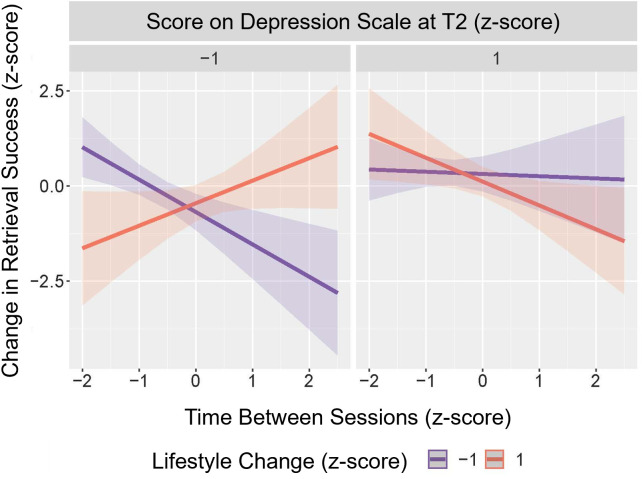
Marginal effects of change in retrieval success model displaying three-way interaction between the time between sessions, lifestyle change, and depression at T2. All values have been converted to z-scores. Shaded areas represent 95% confidence interval. Higher scores on the depression scale indicate a greater degree of depressive symptoms. Results suggest that lifestyle change did not have a substantial influence on the change in retrieval success, with increased time, in participants with higher depression scores (+1 SD). In contrast, for participants with lower depression scores (−1 SD), the predicted change in retrieval success differed substantially with lifestyle change, such that a longer time between T1 and T2 had a more negative effect on retrieval success in individuals with greater reduction in lifestyle engagement.

**Change in retrieval success.** The best-fitting linear regression model of change in retrieval success included the time between sessions, lifestyle change, and depression at T2, with a significant three-way interaction between these variables (influential cases excluded; b = −0.50, 95% CI [−0.87, −0.14], t(41) = 2.79, *p* = .008, partial f^2^ = .19). Visual inspection of the marginal effects (Fig 7) in participants with higher depression scores at T2 (+1 SD) suggests that lifestyle change did not have a substantial influence on the change in retrieval success as the time between sessions increased. This aspect of the results could not be explained by participants with a higher depression score experiencing fewer lifestyle changes due to a less diverse lifestyle to begin with given that lifestyle change (T1-T2 difference score) and depression score were not significantly correlated (r(57) = 0.058; *p* = .66, BF_01_ = 5.61). Furthermore, additional correlation analyses indicated that there was no direct relationship between depression scores at T2 and retrieval success at either timepoint, or the change in retrieval success (Kendall’s Tau: p > .23, BF_01_ > 2.72).

In the model, for participants with lower depression scores (−1 SD), the predicted change in retrieval success differed substantially with lifestyle change. The results suggest that if these participants experienced a more negative change in lifestyle, their retrieval success demonstrated an increasingly negative change as the time between sessions was greater. However, if these participants had experienced a relatively positive change in their lifestyle, then their change in memory success was increasingly positive with an increased time between sessions.

Investigating the three-way interaction (lifestyle change x depression x time between sessions) revealed that in participants with low depression scores (−1 SD), the slope of lifestyle change was substantially different based on the time between testing sessions. In particular, the slope of lifestyle change was borderline non-significant and negative when time between sessions was low (−1 SD; b = −0.61, SE = .24, t(41) = 2.61, p = .05) and significant and positive when the time between sessions was high (+1 SD; b = 0.83, SE = .29, t(41) = 2.90, p = .02). In contrast, when participants had high depression scores (+1 SD) the lifestyle change slope was non-significant regardless of the time between sessions (p > .73). Pairwise slope comparisons indicated a significant difference in lifestyle change slope based on time between sessions (T1-T2) in participants with low depression scores (−1 SD; t(41) = 3.21, p = .02), all other comparisons were non-significant (p > .14). All analyses of lifestyle change slopes were Bonferroni-corrected. Full details of this model are provided in the S1 File and this interaction effect is explored further in the Discussion.

### Experiment 2 discussion

#### Pandemic-related lifestyle changes.

We observed expected declines in lifestyle engagement during the COVID-19 pandemic, as measured using the LEQ, a lifestyle questionnaire that captures activities related to cognitive reserve [23]. Previous studies indicate that loneliness [22] and social activity [21] were particularly impacted during the pandemic. This pattern of results is replicated by the current study where we observed significant declines in social activities (e.g., seeing family and friends) and events of entertainment (e.g., going to the theatre), whereas, many cognitive activities, such as reading and information acquisition, were maintained (Fig 4).

The question of whether a reduction in only social behaviours would lead to cognitive decline has not yet been fully addressed by existing studies, although there is some evidence which suggests that both social and cognitive activity significantly and independently account for variation in cognitive ability [57]. However, when social and cognitive activity were previously included in a model simultaneously, with other lifestyle factors, the social activity estimate changed sign from positive to negative, with one explanation being that social activity devoid of cognitive engagement is not conducive to reserve [57]. This is consistent with another study demonstrating that the association between social activity and multiple cognitive domains was mediated by cognitive activity [58]. Overall, this may indicate that at least some of the benefit of social activity is derived from increased involvement in cognitive activities that accompany them. In the current study, we assessed whether significant social activity reduction impacted cognitive performance, as measured using the LTM task.

#### Memory changes.

Memory was assessed using a LTM cued recall task which allows for the estimation of two distinct memory parameters, retrieval success and precision. There is debate on whether memory is most appropriately characterised by two parameters, with some evidence in support of a single-parameter model of memory [59]. This may be true for short-, but not long-term memory tasks where retrieval success and precision can be differentially sensitive to experimental manipulations [60,61] and engage dissociable brain regions [30,62] suggesting they are at least partially separable. The current study also contained a comparison of memory models, which included a single-parameter model that assumes no guessing occurred, which did not fit the data as well as the two-parameter model. Evidence also suggests retrieval success and precision are differentially impacted by age, with significant declines in precision observed between younger and older adults [32,33,63]. Such findings motivated the hypothesis that these memory features might also be differentially sensitive to the impact of lifestyle changes.

In the current experiment, there was no evidence overall to suggest that lifestyle change during the pandemic substantially influenced memory performance at the group level, using either a correlation analysis, with moderate evidence in favour of no correlation, or modelling including covariates. However, exploratory models considering additional factors with evidenced links to memory performance were also fit separately to memory precision and retrieval success, revealing relationships which are discussed below.

**Precision.** The current experiment identified a significant interaction effect between age, gender, and time point on memory precision. In particular, there was a significant negative effect of age on memory precision in female participants, consistent with prior studies [33]. However, in male participants, there was little impact of age on performance at T1, but at T2 older participants demonstrated higher memory precision. As the influence of age is only apparent at T2, this result could suggest that relatively younger male participants (in their 60s) were impacted more negatively by the pandemic than their older counterparts (in their 70s and 80s). Existing studies do indicate more negative effects in younger and/or middle-aged adults compared with older adults [18,26; although see: 64], although it is unclear why this effect would only be apparent here in males. One possibility is that women had more caring and household responsibilities, even in later life, which may have been exacerbated during the pandemic. Alternatively, the result could be due to selective attrition whereby older male participants who returned at T2 were particularly resilient and high performing. Even though a responder versus non-responder analysis found no differences, with moderate evidence for all variables apart from age and mean absolute error where evidence was anecdotal, it was not possible to account for how people were differently impacted by the pandemic as this data was not available for non-responders.

**Retrieval Success.** Age did not meet the criteria for inclusion in a model for retrieval success, which is consistent with previous research suggesting that it is not as sensitive to age-related differences as precision [33]. Instead, the model included the time between sessions, time point, family history of dementia, and lifestyle. A significant interaction effect between lifestyle and timepoint was only apparent in participants with a family history of dementia. Given the relatively small number of participants in this subgroup, this finding should be interpreted with caution, as it may be susceptible to type I error. Nevertheless, the exacerbated influence of lifestyle on memory success in people at increased risk of dementia is in line with the concept of cognitive reserve [7]. Specifically, Cognitive Reserve theory predicts that lifestyle engagement provides a buffer to the impact of underlying brain changes which are likely to be more substantial in those with increased dementia risk.

Furthermore, the effect of timepoint on retrieval success was more negative in participants with greater lifestyle engagement scores. This pattern of results could be accounted for by those with the highest typical lifestyle engagement experiencing the greatest change to their lifestyle during the pandemic. This may have had a detrimental effect on memory performance via the reduction of cognitive reserve which is proposed to be protective of cognitive performance, particularly in older adults or those with underlying neuropathology [7]. Alternatively, reduced lifestyle engagement might have had a detrimental impact on cognition due to its effects on mental health. The exacerbating effects of the pandemic on mental health issues are well evidenced [18,25,26,65,66], and the importance of mental health in the relationship between lifestyle and memory change is supported by the model-based analysis of change in retrieval success in the current experiment, where the best-fitting model across all participants included the time between sessions, lifestyle change and depression score.

In participants with higher depression scores at T2 (+1 SD), lifestyle change had little influence on the change in retrieval success with increasing time between sessions, whereas, in those with lower depression scores (−1 SD), the predicted change in retrieval success differed substantially with lifestyle change. If participants had experienced a more negative change in lifestyle, their retrieval success also declined with increasing time between sessions. However, if they had experienced a relatively positive change in their lifestyle then their change in memory success was increasingly positive with an increased time between sessions.

An associated decline of depression and cognitive performance during the COVID-19 pandemic was observed in several previous studies [18,24,26], and the general relationship between depression and episodic memory deficits is well-evidenced [67]. A study carried out before the pandemic found evidence to suggest that depression is one factor mediating the relationship between social isolation and cognitive function [68]. The potential biological pathways which may lead to a mediation effect of depression on cognition include reduced hippocampal function, the brain area associated with retrieval success [30], and inflammatory changes [69]. Of note, the depression scores present in the current sample would be considered “subthreshold depression” as they were mostly low across the sample; however, depression-related cognitive deficits have also been found in those with subthreshold symptoms [70]. The current results suggest that the relationship between lifestyle change and change in memory retrieval success varies based on the level of depressive symptoms, which may suggest that when depression is more severe it is the dominant effect on memory, whereas, when depression is milder, other factors such as lifestyle are more impactful. However, as depression was only measured for all participants at T2, and we did not observe a direct association between depression and retrieval success in correlation analyses, further research is required to aid our understanding of this relationship.

Overall, in the current study, we observed an influence of lifestyle on retrieval success whereas none of the best-fitting precision models included lifestyle. As previously mentioned, precision has been shown to be particularly sensitive to age [32,33]. However, there have also been instances where retrieval success was selectively impacted, such as in forgetting over time [60] and in Autism Spectrum Disorder (ASD; [62]). In both cases the impact on retrieval success alongside preserved precision was explained via diminished accessibility at the retrieval stage. Cooper et al., (2017) linked this pattern of behaviour to reductions in both frontal activity and hippocampal connectivity at retrieval and suggested that individuals with ASD were less able to use recollection-based strategies. It has previously been proposed that cognitive reserve is related to effective strategy use [71,72] and has often been linked to frontal regions in older adults [73]. The findings of the current study, whereby reduced engagement in cognitive-reserve-related activities selectively impacted retrieval success, may be accounted for by a reduction in the effective use of recollection-based strategies. However, this account should be explored further in future studies.

**Subjective memory (PRMQ).** The mixed use of subjective and objective memory measures may be one driver of inconsistent previous findings when assessing the impact of pandemic-related restrictions on cognition. Subjective memory decline during the pandemic may be due to reduced activity diversity [18] with the current results finding a significant decline in the number of different activities undertaken in a typical day during the pandemic (Fig 4). Low activity diversity may lead to a lower frequency of memorable events compared with pre-pandemic times [27]. Furthermore, a previous study found that unique events during the COVID-19 pandemic were remembered with more episodic detail than routine events [28]. However, in the current study a subgroup of participants who completed a questionnaire at both time points capturing everyday memory slips, found no change in overall subjective memory performance. Furthermore, no changes were observed in subscores capturing either prospective memory, the ability to act on delayed intentions, or retrospective memory which involves recalling past information. This might also be accounted for by reduced activity diversity during the pandemic, resulting in fewer opportunities for different types of memory slips to occur. Therefore, care must be taken in how subjective memory is assessed and interpreted.

## General discussion

The COVID-19 pandemic had a detrimental impact on many lifestyle factors that have been linked to cognitive reserve. In Experiment 1, a comparison of in-person and online performance in the LTM cued recall task identified comparable results regardless of test environment. This enabled the use of the online task to assess memory performance during the pandemic when in-person testing was severely restricted.

We did not find an overall decrease in memory performance due to pandemic-related restrictions, even though lifestyle activities that may protect against cognitive decline were severely curtailed during that period. These findings help to alleviate concerns raised widely during the pandemic that older adults might be particularly vulnerable to the effects of the restrictions imposed. It may be that the relatively brief nature of the restrictions (lasting several months at a time in the UK) mitigated effects on the older adult group as a whole. However, further analysis revealed that the relationship between lifestyle and retrieval success differed based on depression score and family history of dementia. This suggests that particularly vulnerable members of the older adult population, such as those at higher risk of dementia, may have been disproportionately impacted. For precision, the dominant effect was age, though with different patterns for males and females. Overall, these results provide further evidence of a dissociation between precision and retrieval success with independent variables explaining their between-subject variability and change across time and highlight the importance of a holistic approach to protecting cognition in older adults with consideration of mental health as well as modifiable lifestyle interventions.

## Supporting information

S1 File Supporting Information. (DOCX)
