# Supplementary material for: The impact of lifestyle restrictions on memory in older adults
Source: PLoS One. 2026 Feb 17;21(2):e0342458. doi: 10.1371/journal.pone.0342458 (PMC12912564; doi:10.1371/journal.pone.0342458)
Supplement: S1 File — (DOCX) [file pone.0342458.s001.docx]

**The impact of lifestyle restrictions on memory in older adults**

**Supplementary Materials**

**Model Comparison Approach**

Three models were fit to the response error data to determine whether responses were best represented by a single parameter model reflecting variable memory precision or whether additional response types (e.g. guessing) should be considered (Figure S1). Model 1 assumes that all responses are target-based (von Mises distribution centred on the target value). Model 2 adds a random guessing component (uniform distribution). Lastly, Model 3 also includes non-target responses (von Mises distribution centred on the non-target value). Akaike information criterion (AIC), Bayesian information criterion (BIC) and Deviance information criterion (DIC) were used to determine the best-fitting model for generating memory outcomes.

Figure S1.

*Mixture model components*

**
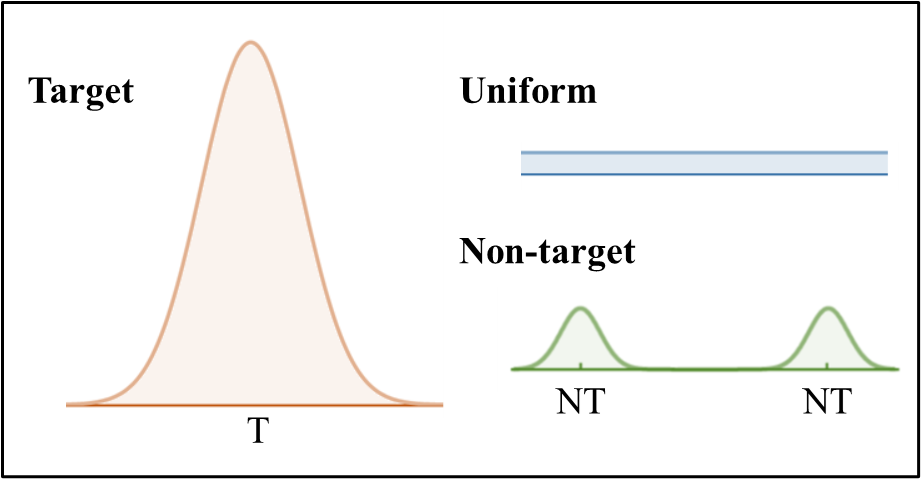
**

**Online Testing: Technological Issues**

An issue was identified in the online task where the target object moved around the circular display at a slower rate during the location response for users with a Macintosh (Mac) operating system. In the samples analysed here there were small numbers of Mac users, therefore, it was not possible to fully assess the impact of this in the current data. However, this was assessed in a large sample of data (N=399) acquired from middle-aged adults for another study and there were no significant effects on memory performance but as expected significantly longer response times for Mac users. In Experiment 1 of the current study, we observed no modality effects on location response times, possibly due to the small number of Mac users in the sample. Nonetheless, future uses of the online task could restrict recruitment to non-Mac users to limit sources of variability, although the data suggests performance is not substantially impacted.

**Experiment 1**

**Model Comparison Results**

Model fit metrics predominantly favoured Model 2 (Table S1) suggesting that in addition to variable memory precision for the target location, response errors sometimes reflected no memory for the target location. In some cases Model 3 was favoured, however, as non-target item response estimates were consistently very low and based on previous findings using this task [1–3], the main analysis used Model 2 (von Mises and Uniform distribution; Figure S1).

Table S1. AIC, BIC and DIC differences between the considered models (group level)

| **Model comparison** | **AIC** | **BIC** | **DIC** | **Best model AIC** | **Best model BIC** | **Best model DIC** |
| --- | --- | --- | --- | --- | --- | --- |
| *Online* |  |  |  |  |  |  |
| Model 2 – Model 1 | -1086.43 | -1078.84 | -1086.33 | Model 2 | Model 2 | Model 2 |
| Model 2 – Model 3 | -1.50 | -9.09 | -0.72 | Model 2 | Model 2 | Model 2 |
| *In Person* |  |  |  |  |  |  |
| Model 2 – Model 1 | -1028.56 | -1021.00 | -1028.52 | Model 2 | Model 2 | Model 2 |
| Model 2 – Model 3 | 6.08 | -1.47 | 5.48 | Model 3 | Model 2 | Model 3 |

**Model-derived guessing threshold**

As found previously [3] it may not be possible to consistently generate robust parameter estimates at the participant level, particularly when participants have fewer trials with target-based responses, as these are the trials used to estimate precision.

To prevent data loss from poor model fits, subject-level outcomes can be generated using a guessing threshold approach adopted from previous studies [2–5]. The mixture model [6] was fit to the group-level data using the BAYSLAB toolbox (<https://bayslab.com/toolbox>) to generate trial-by-trial probabilities that responses were guesses. This was used to determine at which angular distance from the target feature values, responses were less than 5% likely to be memory-based and this distance was used as the guessing threshold. The threshold was based on all trials, across participants and groups, and was therefore robust to issues in model-fitting to participant-level data. Using this threshold, subject-level estimates of retrieval success were calculated as the proportion of responses within the cut-off value and estimates of precision were based on the standard deviation of the error values for responses within the cut-off value (multiplied by minus one so that higher values indicate greater precision).

**Response Times**

Response times ≥3 SDs from the group mean were excluded. These exclusions did not impact the overall conclusions of the response time analyses.

Figure S2

*Response Times by Test Environment*

| 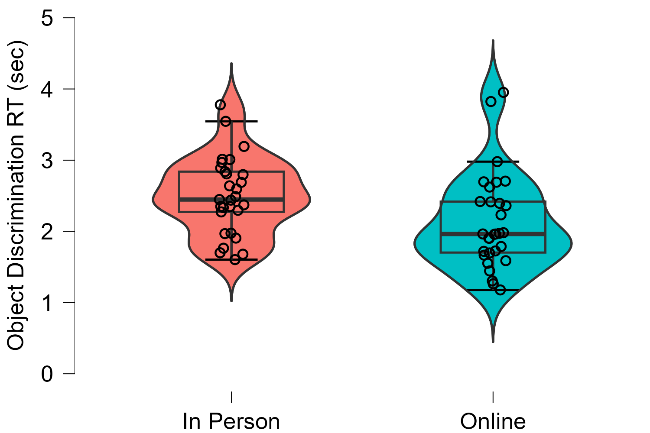 | 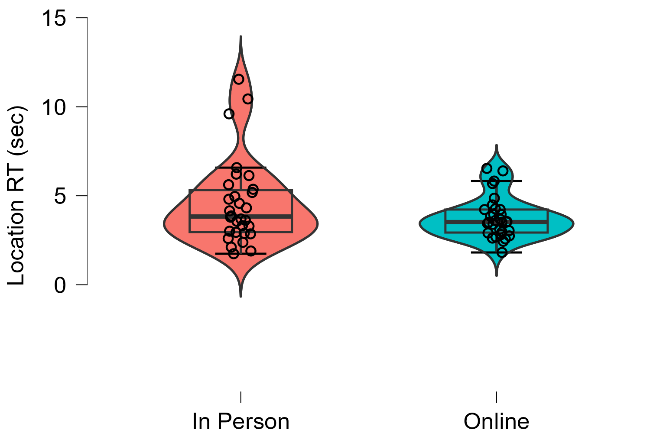 |
| --- | --- |

*Note*. Excludes times >3 SDs from group mean. Plots present the median as the central line, the first and third quartiles as the edges of the box, and the lines indicate the minimum and maximum values (within the range of the first and third quartiles multiplied by 1.5 times the interquartile range) for the (Left) mnemonic discrimination response and the (Right) location response.

***Object Mnemonic Discrimination***

The response times of two participants were excluded (one in-person, one online). Response times for mnemonic discrimination judgements were significantly slower in-person (M=2.51s, SD=0.54) than online (M=2.13s, SD=0.67; *W* = 607.00, *p* = .009). However, the Bayes Factor (*BF_10_*=2.77) indicated only anecdotal evidence in favour of a difference between groups (Figure S2).

***Location Response***

When making location responses, there was no significant difference in response times between test environments (In-person: M=4.57s, SD=2.41, Online: M=3.75s, SD=1.14; *W* = 541.00, *p* = .28) though with only anecdotal evidence in favour of no group difference (*BF_01_* = 2.38).

***Summary***

Mnemonic discrimination responses were significantly slower in person, though the strength of evidence was anecdotal. Location response times were comparable across environments, though again with only anecdotal evidence. Previous studies have found response time differences between task settings [7] though with slower responses online. In the current study, the faster online responses for mnemonic discrimination might have been due to the participants’ motivation to complete the task quickly and the varied hardware used to measure response times, except one would then expect to observe consistent results for the location response. A better explanation may be that the analysis was likely more sensitive to timing changes for the object recognition responses because they were typically fast with less between-subject variation.

**Experiment 2**

**Model Comparison Results**

Only Model 1 and Model 2 were compared for the Korkki et al. (2023) experiments because only one item was displayed, meaning that swap errors could not occur. All model criteria (AIC, BIC and DIC) favoured Model 2 across each baseline study and the T2 online study (Table S2).

Table S2. AIC, BIC and DIC differences between the considered models (group level)

| **Model comparison** | **AIC** | **BIC** | **DIC** | **Best model**  **(AIC/BIC/DIC)** |
| --- | --- | --- | --- | --- |
| *Korkki et al., 2020: Experiment 1* | | | | |
| Model 2 – Model 1 | -878.52 | -870.81 | -878.64 | Model 2 |
| Model 2 – Model 3 | -2.00 | -9.72 | -1.90 | Model 2 |
| *Korkki et al., 2020: Experiment 2* | | | | |
| Model 2 – Model 1 | -412.55 | -405.15 | -412.77 | Model 2 |
| Model 2 – Model 3 | -2.00 | -9.40 | -1.40 | Model 2 |
| *Korkki et al., 2020: Experiment 3* | | | | |
| Model 2 – Model 1 | -499.09 | -491.28 | -499.59 | Model 2 |
| Model 2 – Model 3 | -2.00 | -9.80 | -1.50 | Model 2 |
| *Korkki et al., 2023: inside fMRI* | | | | |
| Model 2 – Model 1 | -565.95 | -558.57 | -566.08 | Model 2 |
| *Korkki et al., 2023: outside fMRI* | | | | |
| Model 2 – Model 1 | -1057.47 | -1049.73 | -1057.42 | Model 2 |
| *Gellersen et al., 2024* | | | | |
| Model 2 – Model 1 | -689.41 | -681.31 | -689.46 | Model 2 |
| Model 2 – Model 3 | -2.00 | -10.11 | -1.72 | Model 2 |
| *Follow-up (T2)* | | | | |
| Model 2 – Model 1 | -1293.43 | -1285.18 | -1293.83 | Model 2 |
| Model 2 – Model 3 | -1.81 | -10.06 | -0.85 | Model 2 |

Table S3*.* T1 Study Design Comparison

Eligibility and exclusion criteria for the original studies are published elsewhere (Gellersen et al., 2024; Korkki et al., 2020, 2023).

| **Experiment** | | **Participants** | **Items in display (tested)** | **Display duration in seconds** | **Features tested (used in current study)** |
| --- | --- | --- | --- | --- | --- |
| Korkki et al., (2020) | Exp 1 | Older participants in this study scored in the healthy range (26 or above) on the Montreal Cognitive Assessment (MoCA), a screening tool for cognitive impairment [10]. | 3 (2) | 9 | Loc (Loc) |
|  | Exp 2 |  | 3 (2) | 12 | Loc, Col, Ori (Loc) |
|  | Exp 3 |  | 3 (3) | 9 | Colour (Col) |
| Korkki et al., (2023) | | All older participants scored within the healthy range (≥ 26) on the MoCA. Of the 51 older adults that completed the study, 21 completed the memory task while undergoing an fMRI scan, the rest took part outside of the scanner. Due to potential performance differences, these participants are considered separately within the current study. | 1 (1) | 5­­ | Loc, Col (Loc) |
| Gellersen et al., (2024) | | Older participants (aged 60+) are considered in the current analysis. This study also involved completion of the Addenbrooke’s Cognitive Examination (ACE; [11]) and older adults’ scores ranged from 85-100 (mean=95.69). | 3 (3) | 8 | Loc (Loc) |

**Lifetime of Experiences Questionnaire (LEQ) scoring procedure**

The scoring procedures for each section are outlined in Table S4 to Table S6. The scoring approach was chosen with the aim of maintaining consistency where possible with the original questionnaire [12].

Table S4*. Scoring procedure for Youth-Specific section of LEQ (Education)*

| **Question** | **Scoring procedure** |
| --- | --- |
|  |  |
| ***Training undertaken in youth (years, FT/PT, % completed) – all scores below multiplied by % of course completed*** | |
| Clerical, administrative, or basic book-keeping training | Scored 4 points if 1- or 2-year course duration, 6 points if >2 years |
| Business Course | Scored 4 points if 1- or 2-year course duration, 6 points if >2 years |
| Trade Apprenticeship | Scored 6 points if 1- or 2-year course duration, 8 points if >2 years |
| Other Technical Course | Scored 8 points |
| College Diploma | Scored 8 points |
| University Undergraduate (3 or 4 year degree) | Scored 10 points |
| University Undergraduate (5 or 6 year degree) | Scored 10 points |
| University Masters | Scored 8 points |
| University PhD/Doctorate | Scored 10 points |
| Other Graduate Course | Scored 4 points per year of study |
| Other courses | Scored 1 point per year of study (includes courses A-level equivalent and above) |

Table S5*. Scoring procedure for Midlife Specific section of LEQ (Occupation)*

| **Question** | **Scoring procedure** |
| --- | --- |
|  |  |
| ***Job classification scored for each five-year interval from age 30 to 65 years:*** | |
| Manager and Admin | 9 points |
| Professional | 8 points |
| Associate Professional | 7 points |
| Tradespersons | 6 points |
| Advanced Clerical | 5 points |
| Intermediate Clerical | 4 points |
| Intermediate Production | 3 points |
| Elementary Clerical | 2 points |
| Labourers | 1 point |
| Homecare | 0 points |
| Nil | 0 points |
| ***Number of people in charge of/ responsible for in each role: scored across each five-year interval from age 30 to 65 years:*** | |
| None/not applicable | 8 points |
| 1-5 people | 16 points |
| 6-10 people | 24 points |
| >10 people | 32 points |

Table S6. *Scoring procedure for Non-Specific section of LEQ (Typical and Pandemic-Related Lifestyle)*

| **Question** | **Scoring procedure** |
| --- | --- |
|  |  |
| How often were you seeing a member of your family or friends during this time? | Never=0 points, less than monthly=1 point, Monthly=2 points, Fortnightly=3 points, Weekly=4 points, Daily=5 points |
| How often were you practicing or playing a musical instrument? |  |
| How often would you practice or develop an artistic pastime (e.g., drawing, painting, sculpture etc.)? |  |
| How often would you take part in sports or activities that were mildly energetic (e.g., walking, woodwork, weeding etc.)? |  |
| How often would you take part in sports or activities that were moderately energetic (e.g., cycling, golf, lawn mowing etc.)? |  |
| How often would you take part in sports or activities that were vigorously energetic (e.g., running, digging, tennis etc.)? |  |
| How often did you read (material of any sort) for more than 20 minutes? |  |
| How often would you practice speaking, reading, writing, or learning a second language? |  |
| Number of continents travelled to in that life-phase | 0 points if 0 continents, 1 point if 1-2 continents, 2 points if 3-4 continents, 3 points if 5 continents, 4 points if 6-7 continents, 5 points if 8-9 continents |
| *How would you spend a typical day? | 0 points if 1-2 activities, 1 point if 3-4, 2 points if 5-6, 3 points if 7, 4 points if 8, 5 points if 9, 6 points if 10, 7 points if 11, 8 points if 12, 9 points if 13, 10 points if >13 |
| *What types of events or entertainment would you undertake in a typical 2-month period? | 1 point if 1-2 events, 2 points if 3-4 events, 3 points if 5 events, 4 if 6-7 events, 5 points if 8-10 events |
| *How would you usually acquire your information about world and national events? | 1 point if 1 source selected, 2 points if 2-3 sources, 3 points if 4 sources, 4 points if 5 sources, 5 points if >5 sources |
| *What kinds of materials were you reading on a regular basis? | 1 point if 1 material, 2 points if 2-3 materials, 3 points if 4 materials, 4 points if 5 materials, 5 points if >5 materials or they select “all of above” |
| *Did you do any charity or volunteer work? If yes, please also indicate in how many types of volunteer work you were involved: | 1 point if 0, 2 points if 1-2, 3 points if 3-4, 4 points if 5-6, 5 points if ≥7 |
| *Were you an active member of any social clubs or groups? If yes, please indicate how many: |  |

***Note*:** incorporates items from late-life specific section of original LEQ (*) to capture additional details about how lifestyle activities were impacted by pandemic-related restrictions. Items on “continents travelled to”, “charity or volunteer work” and “social clubs or groups” were not included in the main analysis due to time frame bias.

**LEQ Age Cut-offs**

The age cut-off for inclusion as an older adult within the current studies (60 years) was different to the late-life threshold in the LEQ (65 years). Therefore, participants under the age of 66 years at T2 did not complete the late-life section of the LEQ. Furthermore, participants aged 66 in 2021 had not experienced much, if any, time over the age of 65 years without pandemic-related restrictions. The responses on the late-life (non-specific) section of the LEQ were considered the participants’ “typical” lifestyle in the current analysis for most participants, however, for the reasons mentioned above, this was not possible for participants under the age of 67 years (eight participants). Therefore, their responses on the mid-life (non-specific) section of the LEQ were used as their “typical” lifestyle scores instead.

**Best-Fitting Models**

**Lifestyle and Memory**

Table S7: Summary of the Best Fitting Model for Change in Retrieval Success

|  | **Change in Retrieval Success** | | |
| --- | --- | --- | --- |
| *Predictors* | *Estimates* | *CI* | *p* |
| (Intercept) | -0.21 | -0.42 – -0.01 | **0.043** |
| Time Between Sessions | -0.34 | -0.55 – -0.12 | **0.003** |
| Observations | 55 | | |
| R^2^ / R^2^ adjusted | 0.159 / 0.143 | | |

Table S8: Summary of the Best Fitting Model for Absolute Precision

|  | **Absolute Precision** | | |
| --- | --- | --- | --- |
| *Predictors* | *Estimates* | *CI* | *p* |
| (Intercept) | -0.12 | -0.44 – 0.19 | 0.430 |
| Time point | 0.05 | -0.09 – 0.19 | 0.507 |
| Age (T1) | 0.08 | -0.13 – 0.28 | 0.449 |
| Gender (Female) | 0.11 | -0.09 – 0.31 | 0.274 |
| Time point * Age | 0.14 | -0.00 – 0.28 | 0.057 |
| Time point * Gender | -0.01 | -0.15 – 0.13 | 0.879 |
| Age * Gender | -0.33 | -0.52 – -0.13 | **0.001** |
| Time point * Age * Gender | -0.19 | -0.33 – -0.05 | **0.010** |
| **Random Effects** | | | |
| σ^2^ | 0.50 | | |
| τ_00_ _IDF_ | 0.22 | | |
| τ_00_ _BaselineExpF_ | 0.09 | | |
| ICC | 0.38 | | |
| N _IDF_ | 59 | | |
| N _BaselineExpF_ | 6 | | |
| Observations | 113 | | |
| Marginal R^2^ / Conditional R^2^ | 0.153 / 0.477 | | |

Table S9: Summary of the Best Fitting Model for Absolute Precision (Females Only)

|  | **Absolute Precision** | | | | |
| --- | --- | --- | --- | --- | --- |
| *Predictors* | *Estimates* | *CI* | *Statistic* | *p* | *df* |
| (Intercept) | 0.01 | -0.22 – 0.25 | 0.12 | 0.901 | 71.00 |
| Age (T1) | -0.29 | -0.53 – -0.05 | -2.41 | **0.019** | 71.00 |
| **Random Effects** | | | | | |
| σ^2^ | 0.47 | | | | |
| τ_00_ _IDF_ | 0.31 | | | | |
| ICC | 0.40 | | | | |
| N _IDF_ | 39 | | | | |
| Observations | 75 | | | | |
| Marginal R^2^ / Conditional R^2^ | 0.096 / 0.456 | | | | |

Table S10: Summary of the Best Fitting Model for Absolute Precision (Males Only)

|  | **MTK** | | |
| --- | --- | --- | --- |
| *Predictors* | *Estimates* | *CI* | *p* |
| (Intercept) | -0.23 | -0.55 – 0.10 | 0.162 |
| Time point | 0.06 | -0.17 – 0.30 | 0.591 |
| Age (T1) | 0.36 | 0.03 – 0.70 | **0.033** |
| Time point * Age | 0.33 | 0.09 – 0.57 | **0.008** |
| **Random Effects** | | | |
| σ^2^ | 0.50 | | |
| τ_00_ _IDF_ | 0.24 | | |
| ICC | 0.32 | | |
| N _IDF_ | 20 | | |
| Observations | 38 | | |
| Marginal R^2^ / Conditional R^2^ | 0.248 / 0.490 | | |

Table S11: Summary of the Best Fitting Model for Absolute Retrieval Success (Excluding Influential Cases)

|  | **Absolute Retrieval Success** | | |
| --- | --- | --- | --- |
| *Predictors* | *Estimates* | *CI* | *p* |
| (Intercept) | 0.18 | -0.12 – 0.49 | 0.237 |
| Time point (TP) | -0.18 | -0.30 – -0.06 | **0.003** |
| Time Between | -0.07 | -0.47 – 0.34 | 0.742 |
| Lifestyle | -0.00 | -0.25 – 0.25 | 0.975 |
| Family History of Dementia (FHD) | 0.04 | -0.26 – 0.35 | 0.779 |
| TP * Time Between | -0.27 | -0.39 – -0.14 | **<0.001** |
| TP * Lifestyle | -0.05 | -0.17 – 0.07 | 0.368 |
| TP * FHD | -0.08 | -0.20 – 0.04 | 0.176 |
| Time Between * Lifestyle | -0.02 | -0.32 – 0.29 | 0.907 |
| Time Between * FHD | -0.05 | -0.45 – 0.35 | 0.802 |
| Lifestyle * FHD | -0.03 | -0.28 – 0.23 | 0.830 |
| (TP * Time Between) * Lifestyle | -0.12 | -0.24 – 0.00 | 0.058 |
| TP * Time Between * FHD | -0.11 | -0.24 – 0.01 | 0.078 |
| TP * LS * FHD | -0.22 | -0.34 – -0.10 | **0.001** |
| Time Between * Lifestyle * FHD | -0.11 | -0.41 – 0.20 | 0.485 |
| **Random Effects** | | | |
| σ^2^ | 0.14 | | |
| τ_00_ _IDF_ | 0.65 | | |
| ICC | 0.82 | | |
| N _IDF_ | 52 | | |
| Observations | 99 | | |
| Marginal R^2^ / Conditional R^2^ | 0.080 / 0.834 | | |

Table S12: Best Fitting Model for Absolute Retrieval Success (Family History of Dementia)

|  | **Absolute Retrieval Success** | | |
| --- | --- | --- | --- |
| *Predictors* | *Estimates* | *CI* | *p* |
| (Intercept) | 0.25 | -0.22 – 0.72 | 0.281 |
| Time point (TP) | -0.19 | -0.34 – -0.05 | **0.012** |
| Time Between | -0.09 | -0.75 – 0.56 | 0.766 |
| Lifestyle | 0.05 | -0.31 – 0.40 | 0.790 |
| TP * Time Between | -0.25 | -0.38 – -0.12 | **0.001** |
| TP * Lifestyle | -0.23 | -0.37 – -0.10 | **0.002** |
| Time Between * Lifestyle | -0.09 | -0.55 – 0.37 | 0.695 |
| **Random Effects** | | | |
| σ^2^ | 0.06 | | |
| τ_00_ _IDF_ | 0.50 | | |
| ICC | 0.89 | | |
| N _IDF_ | 15 | | |
| Observations | 27 | | |
| Marginal R^2^ / Conditional R^2^ | 0.123 / 0.906 | | |

Table S13: Best Fitting Model for Absolute Retrieval Success (No Family History of Dementia)

|  | **Absolute Retrieval Success** | | |
| --- | --- | --- | --- |
| *Predictors* | *Estimates* | *CI* | *p* |
| (Intercept) | 0.14 | -0.16 – 0.43 | 0.356 |
| Time point (TP) | -0.10 | -0.20 – 0.01 | 0.066 |
| Time Between | -0.03 | -0.33 – 0.27 | 0.849 |
| Lifestyle | -0.01 | -0.28 – 0.27 | 0.967 |
| TP * Time Between | -0.16 | -0.27 – -0.05 | **0.005** |
| TP * Lifestyle | 0.16 | -0.00 – 0.31 | 0.051 |
| Time Between * Lifestyle | 0.10 | -0.18 – 0.39 | 0.472 |
| **Random Effects** | | | |
| σ^2^ | 0.18 | | |
| τ_00_ _IDF_ | 0.70 | | |
| ICC | 0.79 | | |
| N _IDF_ | 37 | | |
| Observations | 72 | | |
| Marginal R^2^ / Conditional R^2^ | 0.054 / 0.806 | | |

Table S14: Summary of the Best Fitting Model for Change in Retrieval Success (Excluding Influential Cases)

|  | **Change in Retrieval Success** | | |
| --- | --- | --- | --- |
| *Predictors* | *Estimates* | *CI* | *p* |
| (Intercept) | -0.18 | -0.36 – 0.00 | 0.056 |
| Time Between | -0.24 | -0.43 – -0.04 | **0.021** |
| Lifestyle Change (LC) | 0.01 | -0.19 – 0.21 | 0.937 |
| Depression score (Depression) | 0.39 | 0.16 – 0.62 | **0.002** |
| Time Between * LC | 0.22 | -0.03 – 0.47 | 0.087 |
| Time Between * Depression | -0.11 | -0.40 – 0.19 | 0.466 |
| LC * Depression | -0.11 | -0.38 – 0.16 | 0.426 |
| (Time Between * LC) * Depression | -0.50 | -0.87 – -0.14 | **0.008** |
| Observations | 49 | | |
| R^2^ / R^2^ adjusted | 0.360 / 0.251 | | |

**Sensitivity Analyses**

Sensitivity analyses were as follows:

1. Excluding participants who took part in the Gellersen et al., (2024) baseline study. This baseline study was most similar in design to the online experiment and was undertaken only shortly before the pandemic. It therefore may be particularly susceptible to practice effects.
2. A sensitivity analysis of possible exclusion criteria was conducted by excluding participants at T2 who had reported prior mild COVID-19 symptoms or current anxiety or depression (or taking antidepressants regularly).
3. The effect of using the midlife section of the questionnaire for some participants’ “typical lifestyle” score is explored in the sensitivity analysis by excluding these participants from the lifestyle analysis (their memory data is still used when calculating z-scores).

*Did Pandemic-related Lifestyle Changes Influence Memory Performance?*

***Memory Precision:***

1. When excluding participants who took part in the Gellersen et al., (2024) baseline study all correlations remained non-significant. Furthermore, the model of change in memory precision was only improved by the addition of age which had a significant positive main effect (b = 0.51, 95% CI [0.16, 0.87], t(29) = 2.95, p = .006, partial f2 = .30).
2. Excluding participants who reported a current neuropsychiatric condition, taking antidepressants, or prior mild COVID-19 symptoms did not impact the results obtained with the full sample.
3. Excluding participants under the age of 67 years did not impact the results obtained with the full sample.

***Retrieval Success:***

1. Excluding participants who took part in the Gellersen et al., (2024) baseline study did not impact the results obtained with the full sample.
2. When excluding participants who reported a current neuropsychiatric condition, taking antidepressants regularly, or prior mild COVID-19 symptoms there was a significant correlation between change in lifestyle and change in retrieval success in participants above the median LIBRA score (*r*(14) = 0.574; p = 0.02) however, this did not remain significant following multiple comparison correction. The results of the linear regression analysis were not impacted by participant exclusions.
3. Excluding participants under the age of 67 years did not impact the results obtained with the full sample.

*Do Other Factors Account for Memory Performance Across Time-Points?*

***Memory Precision:***

1. Excluding participants who took part in the Gellersen et al., (2024) baseline study did not impact the overall results of the analysis.
2. When excluding participants who reported a current neuropsychiatric condition, reported taking antidepressants, or prior mild COVID-19 symptoms the best-fitting model again included age, time-point and gender, however, this model only substantially improved upon model fit metrics once the time-between-sessions variable was excluded.
3. Finally, participants under the age of 67 years were excluded. Here, as above, only once the time-between-sessions variable was excluded did the best-fitting model again include age, time-point and gender.

***Change in Memory Precision:***

1. When excluding participants who took part in the Gellersen et al., (2024) baseline study, the best fitting model included age and gender similar to the main analysis of absolute precision. Here, the best-fitting model was based on R-squared as two models improved upon the base model fit when conducting model comparisons based on AIC and LRT. The model was further improved by removing the time between sessions term from the model, again consistent with the main analysis.

Table S15: Summary of the Best Fitting Model for Change in Precision (Excluding participants from Gellersen et al., (2024))

|  | **Change in Precision** | | |
| --- | --- | --- | --- |
| *Predictors* | *Estimates* | *CI* | *p* |
| (Intercept) | 0.10 | -0.18 – 0.39 | 0.473 |
| Age | 0.59 | 0.27 – 0.90 | **0.001** |
| Gender | 0.24 | -0.04 – 0.53 | 0.093 |
| Age * Gender | -0.49 | -0.80 – -0.17 | **0.004** |
| Observations | 32 | | |
| R^2^ / R^2^ adjusted | 0.419 / 0.357 | | |

1. When excluding participants who reported a current neuropsychiatric condition, taking antidepressants, or prior mild COVID-19 symptoms the best-fitting model included occupation score and anxiety. But once influential cases were removed there were no significant effects.
2. Finally, participants under the age of 67 years were excluded. Here, the best-fitting model included time between sessions, gender and anxiety. But once influential cases were removed there were no significant effects.

***Retrieval Success:***

1. Excluding participants who took part in the Gellersen et al., (2024) baseline study, did not impact the overall results of the analysis.
2. Excluding participants who reported a current neuropsychiatric condition, taking antidepressants or prior mild COVID-19 symptoms, did not impact the overall results of the analysis.
3. When participants under the age of 67 years were excluded, the best-fitting model incorporated timepoint, gender and occupation score. A higher occupation score was associated with greater retrieval success, particularly at T2, but only for male participants.

Table S16: Summary of the Best Fitting Model for Absolute Retrieval Success (Excluding participants under the age of 67 years)

|  | **Change in Retrieval Success** | | |
| --- | --- | --- | --- |
| *Predictors* | *Estimates* | *CI* | *p* |
| (Intercept) | -0.37 | -1.04 – 0.31 | 0.286 |
| Time point (TP) | -0.40 | -0.70 – -0.11 | **0.008** |
| Gender | 0.66 | -0.02 – 1.33 | 0.058 |
| Occupation | 0.67 | -0.07 – 1.40 | 0.074 |
| TP * Gender | 0.35 | 0.05 – 0.64 | **0.022** |
| TP * Occupation | 0.30 | -0.01 – 0.62 | 0.060 |
| Gender * Occupation | -0.48 | -1.21 – 0.25 | 0.194 |
| (TP * Gender) * Occupation | -0.32 | -0.63 – -0.00 | **0.049** |
| **Random Effects** | | | |
| σ^2^ | 0.24 | | |
| τ_00_ _IDF_ | 0.54 | | |
| ICC | 0.69 | | |
| N _IDF_ | 46 | | |
| Observations | 90 | | |
| Marginal R^2^ / Conditional R^2^ | 0.112 / 0.725 | | |

***Change in Retrieval Success:***

1. When excluding participants who took part in the Gellersen et al., (2024) baseline study, the best fitting model included the time between sessions, depression score and family history of dementia. However, once influential cases were removed there were no significant effects.
2. When excluding participants who reported a current neuropsychiatric condition, taking antidepressants, or prior mild COVID-19 symptoms the best fitting model included the time between sessions, depression score and family history of dementia. However, once influential cases were removed there were no significant effects.
3. Finally, participants under the age of 67 years were excluded, here none of the models fully met the criteria (LRT, AIC) to be considered an improvement upon the base model which included just the time between sessions.

**References**

1. Korkki SM, Richter FR, Jeyarathnarajah P, Simons JS. Healthy Ageing Reduces the Precision of Episodic Memory Retrieval. Psychol Aging. 2020. doi:10.1037/pag0000432

2. Richter FR, Cooper RA, Bays PM, Simons JS. Distinct neural mechanisms underlie the success, precision, and vividness of episodic memory. eLife. 2016;5. doi:10.7554/eLife.18260

3. Gellersen HM, Coughlan G, Hornberger M, Simons JS. Memory precision of object-location binding is unimpaired in APOE ε4-carriers with spatial navigation deficits. 2021. doi:10.1101/2020.12.18.423245

4. Cooper RA, Ritchey M. Cortico-hippocampal network connections support the multidimensional quality of episodic memory. Irish M, Colgin L, editors. eLife. 2019;8: e45591. doi:10.7554/eLife.45591

5. Korkki SM, Richter FR, Simons JS. Hippocampal–Cortical Encoding Activity Predicts the Precision of Episodic Memory. J Cogn Neurosci. 2021;33: 2328–2341. doi:10.1162/jocn_a_01770

6. Bays PM, Catalao RF, Husain M. The precision of visual working memory is set by allocation of a shared resource. J Vis. 2009;9: 7–7.

7. Backx R, Skirrow C, Dente P, Barnett JH, Cormack FK. Comparing Web-Based and Lab-Based Cognitive Assessment Using the Cambridge Neuropsychological Test Automated Battery: A Within-Subjects Counterbalanced Study. J Med Internet Res. 2020;22: e16792. doi:10.2196/16792

8. Korkki SM, Richter FR, Gellersen HM, Simons JS. Reduced memory precision in older age is associated with functional and structural differences in the angular gyrus. Neurobiol Aging. 2023;129: 109–120. doi:10.1016/j.neurobiolaging.2023.04.009

9. Gellersen HM, McMaster J, Abdurahman A, Simons JS. Demands on Perceptual and Mnemonic Fidelity Are a Key Determinant of Age-Related Cognitive Decline Throughout the Lifespan. J Exp Psychol Gen. 2024;153: 200–223. doi:10.1037/xge0001476

10. Nasreddine ZS, Phillips NA, Bédirian V, Charbonneau S, Whitehead V, Collin I, et al. The Montreal Cognitive Assessment, MoCA: A Brief Screening Tool For Mild Cognitive Impairment. J Am Geriatr Soc. 2005;53: 695–699. doi:10.1111/j.1532-5415.2005.53221.x

11. Mioshi E, Dawson K, Mitchell J, Arnold R, Hodges JR. The Addenbrooke’s Cognitive Examination Revised (ACE-R): a brief cognitive test battery for dementia screening. Int J Geriatr Psychiatry. 2006;21: 1078–1085. doi:10.1002/gps.1610

12. Valenzuela M, Sachdev P. Assessment of complex mental activity across the lifespan: development of the Lifetime of Experiences Questionnaire (LEQ). Psychol Med. 2007;37: 1015–1025. doi:10.1017/S003329170600938X
